# Short Acquisition Time Super-Resolution Ultrasound Microvessel Imaging via Microbubble Separation

**DOI:** 10.1038/s41598-020-62898-9

**Published:** 2020-04-07

**Authors:** Chengwu Huang, Matthew R. Lowerison, Joshua D. Trzasko, Armando Manduca, Yoram Bresler, Shanshan Tang, Ping Gong, U-Wai Lok, Pengfei Song, Shigao Chen

**Affiliations:** 1 Department of Radiology, Mayo Clinic College of Medicine and Science, Mayo Clinic, Rochester, MN USA; 2 Beckman Institute, University of Illinois at Urbana-Champaign, Urbana, IL USA; 3 Department of Electrical and Computer Engineering, University of Illinois at Urbana-Champaign, Urbana, IL USA; 4 Department of Physiology and Biomedical Engineering, Mayo Clinic College of Medicine and Science, Rochester, MN USA; 5 Coordinated Science Laboratory, University of Illinois at Urbana-Champaign, Urbana, IL USA

**Keywords:** Cancer imaging, Preclinical research, Electrical and electronic engineering

## Abstract

Super-resolution ultrasound localization microscopy (ULM), based on localization and tracking of individual microbubbles (MBs), offers unprecedented microvascular imaging resolution at clinically relevant penetration depths. However, ULM is currently limited by the requirement of dilute MB concentrations to ensure spatially sparse MB events for accurate localization and tracking. The corresponding long imaging acquisition times (tens of seconds or several minutes) to accumulate sufficient isolated MB events for full reconstruction of microvasculature preclude the clinical translation of the technique. To break this fundamental tradeoff between acquisition time and MB concentration, in this paper we propose to separate spatially overlapping MB events into sub-populations, each with sparser MB concentration, based on spatiotemporal differences in the flow dynamics (flow speeds and directions). MB localization and tracking are performed for each sub-population separately, permitting more robust ULM imaging of high-concentration MB injections. The superiority of the proposed MB separation technique over conventional ULM processing is demonstrated in flow channel phantom data, and in the chorioallantoic membrane of chicken embryos with optical imaging as an *in vivo* reference standard. Substantial improvement of ULM is further demonstrated on a chicken embryo tumor xenograft model and a chicken brain, showing both morphological and functional microvasculature details at super-resolution within a short acquisition time (several seconds). The proposed technique allows more robust MB localization and tracking at relatively high MB concentrations, alleviating the need for dilute MB injections, and thereby shortening the acquisition time of ULM imaging and showing great potential for clinical translation.

## Introduction

Super-resolution ultrasound localization microscopy (ULM), the acoustic analogue to optical sub-diffraction-limit techniques such as PALM^1^ and STORM^2^, is a next-generation ultrasound imaging modality that relies on the localization and tracking of individual contrast microbubbles (MBs) in vasculature. Contrast MBs can be considered as acoustic point scatterers, since they are substantially smaller than the typical ultrasound wavelength, but also provide strong acoustic backscatter, permitting the application of signal processing methods that can overcome the inherent compromise between ultrasound frequency and attenuation. ULM has been shown by numerous research groups^3–18^ to break the diffraction limit of conventional ultrasound, resulting in an approximately tenfold improvement in imaging resolution while maintaining imaging penetration depth. Furthermore, as an ultrasound-based imaging modality, ULM is safe, non-invasive, low-cost, and lacks ionizing radiation, thereby promising great clinical significance by providing capillary-level imaging resolution at acoustic-level imaging penetrations. As such, ULM has rapidly gained traction and is applicable to a wide spectrum of preclinical and clinical applications, including the diagnosis and characterization of cancer^4,8^, cardiovascular disease^19^, and the monitoring of neurological activity^20^.

However, ULM is currently challenged by technical and clinical difficulties including long data acquisition times, low temporal resolution, limited clinical dosages of contrast MBs, and inaccurate localization and tracking of MBs due to ultrasound noise and phase aberration^5^. The successful reconstruction of microvasculature via ULM requires an adequate number of MBs in circulation to fully traverse all vasculature of interest. However, MBs must also be sufficiently spatially isolated to allow for accurate and unequivocal localization of signal centroids—thus, poorly isolated MB centroids are typically excluded from analysis. This represents a challenging trade-off for imaging biologically relevant vascular beds: The imaging of small vessels, which typically have slower flow speeds, benefits from higher MB concentrations that increase the probability of MB traversal; however, this is at the expense of more MB signal overlap in larger vessels. Lowering the concentration of MBs by dilution will result in sparser signal events but will also significantly increase the data acquisition time needed to reconstruct a vascular bed. A long acquisition time is challenging to implement *in vivo* as both tissue- and operator-induced motion will degrade the final super-resolution image. The dilution method also requires a constant infusion of MBs or multiple bolus injections, both of which are challenging in a clinical setting. Phase-change droplets, which can change phase from liquid to gas when activated by ultrasound, have been utilized as a contrast agent for ULM and have recently demonstrated the potential for real-time super-resolution imaging *in vitro*^15,18^. Distinct from clinically used MBs, the droplet-based super-resolution method can sparsely activate droplets for localization, allowing for high droplet concentrations, and does not necessarily require frame-to-frame droplet flow for contrast-agent detection. However, further *in vivo* evaluation and validation may be required for this technique.

To break this tradeoff between acquisition time and MB concentration, we propose leveraging the spatiotemporal characteristics of MBs in circulation to improve the performance of MB localization and tracking. Spatiotemporal filtering (e.g.: SVD filtering) is already extensively used in ultrasound blood flow imaging and ULM pre-processing, as a clutter filter to separate MB signals from tissue background. We propose an additional step of signal post-processing to separate MBs into sub-populations based on differences in spatiotemporal flow dynamics, such as movement speed, flow direction, and signal decorrelation. Although MB signals often spatially overlap over the course of an injection bolus, they may be still separable through the judicious use of spatiotemporal filtering. This paper focuses on the use of 3D conical filters in the Fourier domain for detecting and extracting signal differences to separate MB datasets into distinct subsets, each of which can be processed independently. We apply this technique to MB signal acquisitions taken from the chorioallantoic membrane (CAM) of chicken embryos and use optical imaging as validation. We posit that this type of filtering increases the possibility of observing isolated MBs for high-concentration injections and therefore alleviates the need for MB dilution; as a result, it also shortens the corresponding long acquisition time, which is critical for the clinical translation of ULM.

## Results

### Microbubble separation processing splits data based on flow dynamics

**Figure 1:**
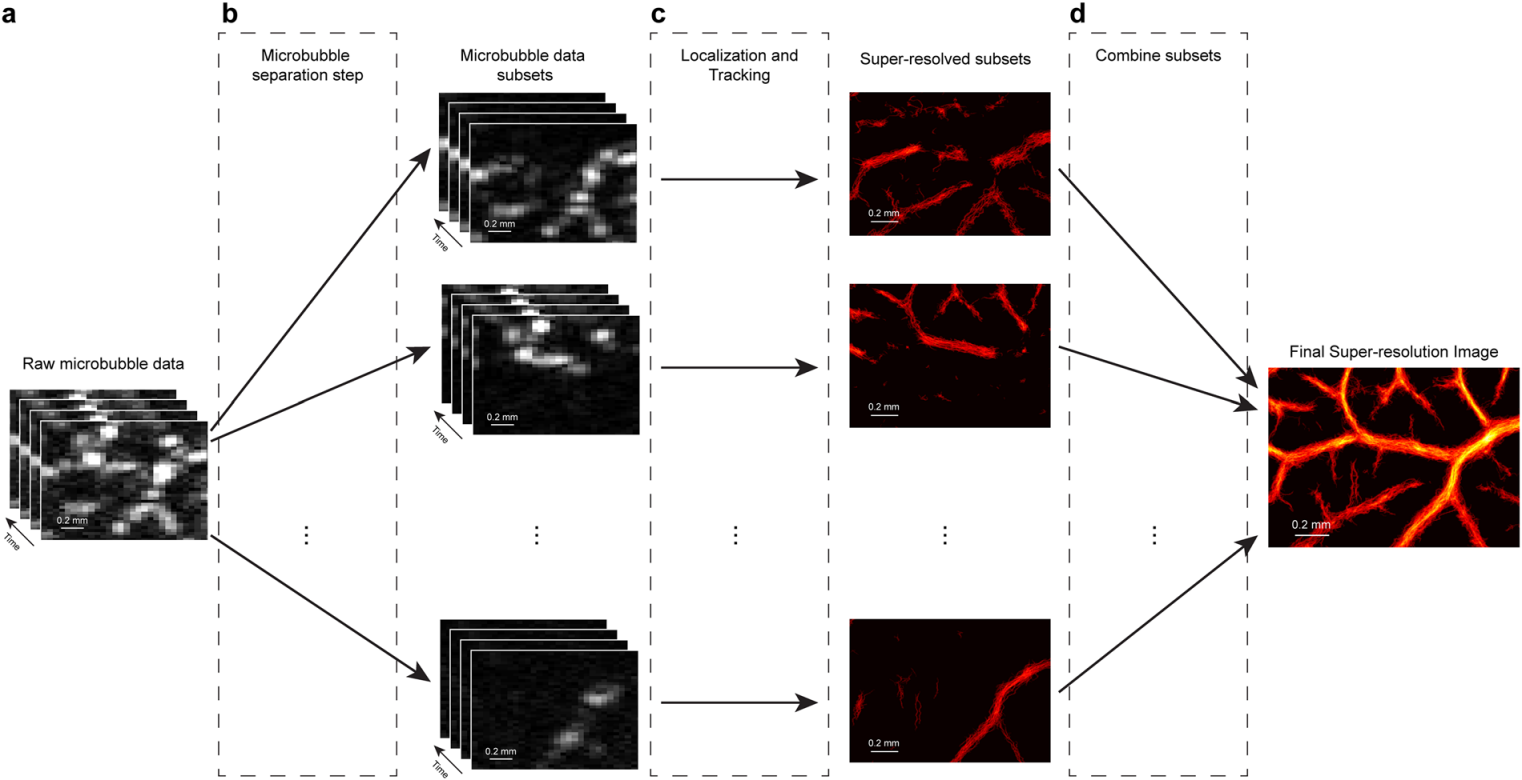
Principle of microbubble separation based super-resolution imaging. (**a**) The original spatiotemporal microbubble data after tissue clutter filtering. (**b**) A 3D Fourier transform separates microbubble subpopulations on the basis of their speed and flow direction, resulting in datasets with sparser microbubble concentration. (**c**) Each data-subset undergoes independent microbubble localization and tracking. (**d**) The final super-resolution ULM image is generated by combining signals from all of the subset data.

The proposed MB separation method divides an original, densely populated MB dataset into subsets of data with a sparser effective MB concentration for more robust localization and tracking. This is demonstrated in the workflow diagram presented in Fig. 1, with additional details provided in the Methods section. When applied to contrast-enhanced images taken from the CAM of chicken embryos, it was found that the technique would separate surface vasculature on the basis of movement speed, flow direction, and decorrelation of MB signals. This led to the generation of super-resolved images that capture different orders of vasculature which can then be combined into a final, high-quality, super-resolved vascular image.

### Flow channel phantom validates microbubble separation processing

**Figure 2:**
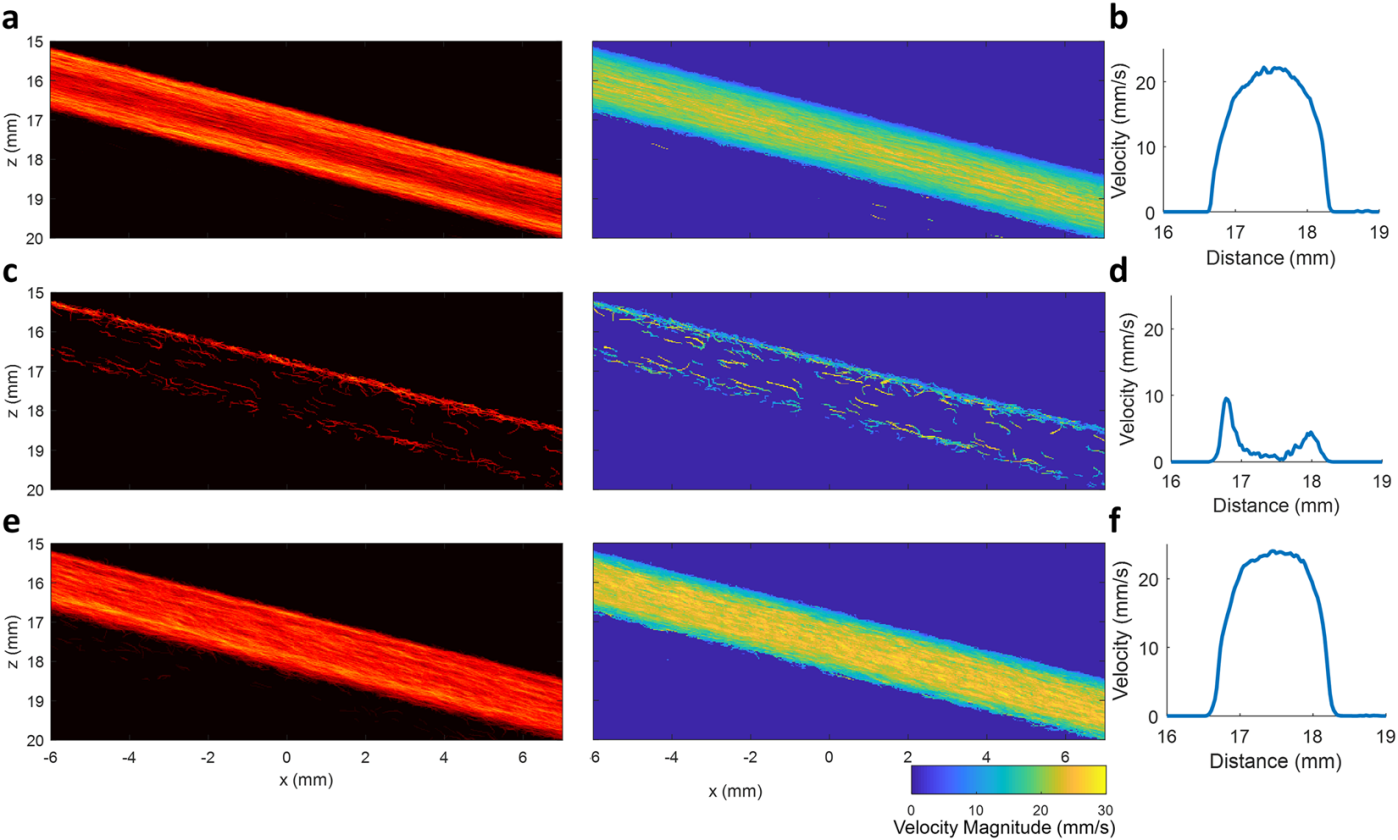
Flow channel phantom validates the proposed method. (**a**) Reference ULM intensity and velocity images of the flow channel phantom that were generated by combining eight individually processed ULM images from separate acquisitions at a low microbubble concentration (a total of four different flow speeds and two different flow directions). (**b**) The combined ULM flow channel image demonstrated a well-defined parabolic flow profile. (**c**) Conventional ULM image processing performed on a high microbubble concentration dataset did not result in an adequate number of high-confidence microbubble events to populate the vessel lumen and (**d**) did not produce a meaningful flow profile. (**e**) Performing microbubble separation on the high microbubble concentration dataset allowed for accurate reconstruction and filling of the vessel lumen and (**f**) could recover a parabolic flow profile.

Separate ultrasound datasets were acquired from a flow channel phantom running at a constant flow rate with a relatively low concentration of MBs (a 2000x dilution of the original solution of Bracco Lumason). A total of four different flow rates (1.89 ml/min, 2.83 ml/min, 3.77 ml/min and 4.71 ml/min) and two different flow directions were combined frame-by-frame to generate a synthetic ultrasound data set. This combined data set was used to test the performance of the MB separation technique, as the data set represents a complicated flow scenario where MBs are flowing at different speeds and directions simultaneously. To serve as a reference standard, each individual low-concentration MB dataset was processed using conventional ULM super-resolution processing (a representative example is shown in Fig. 2a). This yielded a well-characterized parabolic flow profile (Fig. 2b), which is expected from fully-developed flow through a flow channel. In contrast, directly applying conventional super-resolution processing to the synthetically combined dataset (Fig. 2c) does not provide an adequate number of high-confidence MB events to populate the vessel lumen. This result was expected, as MB signals often overlap at high concentrations, causing the identification of MB centroids to be inaccurate. Furthermore, individual MB tracking is also challenging when there are neighboring MBs moving in opposite directions within the same flow channel, which further reduces the number of detected MB trajectories. As a result, conventional ULM imaging was unable to construct a meaningful flow profile (Fig. 2d) from the synthetically combined ultrasound dataset. Only the luminal space adjacent to the flow channel wall was detected as MB flow, which was likely due to the lower concentration and correspondingly sparser MB events.

MB signals with different velocity ranges and/or flow directions within the synthetic dataset could be separated into different subsets by applying the proposed algorithm. The MB signals within these data subsets could be more accurately localized and tracked due to the lower effective concentration (i.e. sparser events) and more consistent flow direction. ULM was performed on each data subset individually, producing separate super-resolution images (Sup Fig. 1). A final ULM image could then be generated by combining all of the image subsets (Fig. 2e). Figure 2e demonstrates that a sufficient number of MB events were detected to fully populate the entire vessel lumen and that a parabolic velocity profile (Fig. 2f) could be accurately recovered from this dataset. This illustrates that the proposed MB separation method can achieve superior performance for ULM at high MB concentrations and for complex hemodynamic situations.

**Figure 3:**
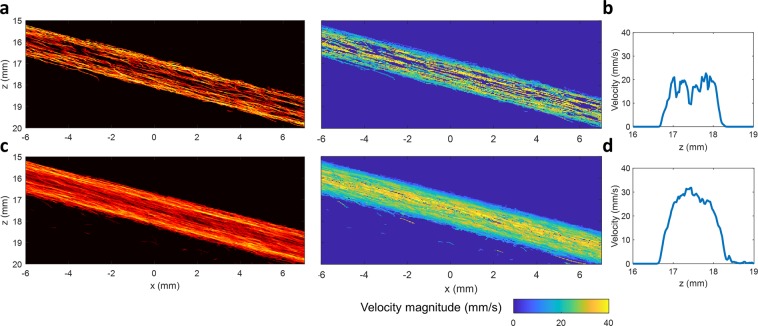
ULM images obtained from one individual flow channel data. (**a**) ULM intensity and velocity images of the flow channel obtained without MB separation. (**b**) The flow speed profile obtained without MB separation. (**c**) ULM intensity and velocity images of the same flow channel data obtained with MB separation method. (**d**) Well-developed parabolic flow speed profile obtained with the MB separation method. For one individual flow channel data, different flow velocities inherently exist along the radial direction. A stack of 2000 frames was used to produce the ULM image for this figure. The proposed method provided a higher number of localized MBs, permitting a denser population of the lumen of the flow channel.

It should be noted that generating synthetic data by adding up different individual datasets to simulate high MB concentration does not represent a true physiological blood flow situation. For a more realistic situation, the proposed method was also applied to one individual flow channel data, where different flow velocities exist naturally along the radial direction. The results are shown in Fig. 3, which again demonstrates that a larger number of MBs were detected by the proposed MB separation method (Fig. 3c), allowing for a more densely populated vessel lumen when compared with the conventional non-separation method (Fig. 3a). This is particularly apparent for the center region of the channel, where the blood flow speed is highest. A well-developed parabolic speed profile (Fig. 3d) could be reconstructed by the proposed method, while the non-separation method did not provide a sufficient number of detected MBs to fully recover a parabolic speed profile (Fig. 3b).

### *In vivo* microbubble separation is validated with optical microscopy

**Figure 4:**
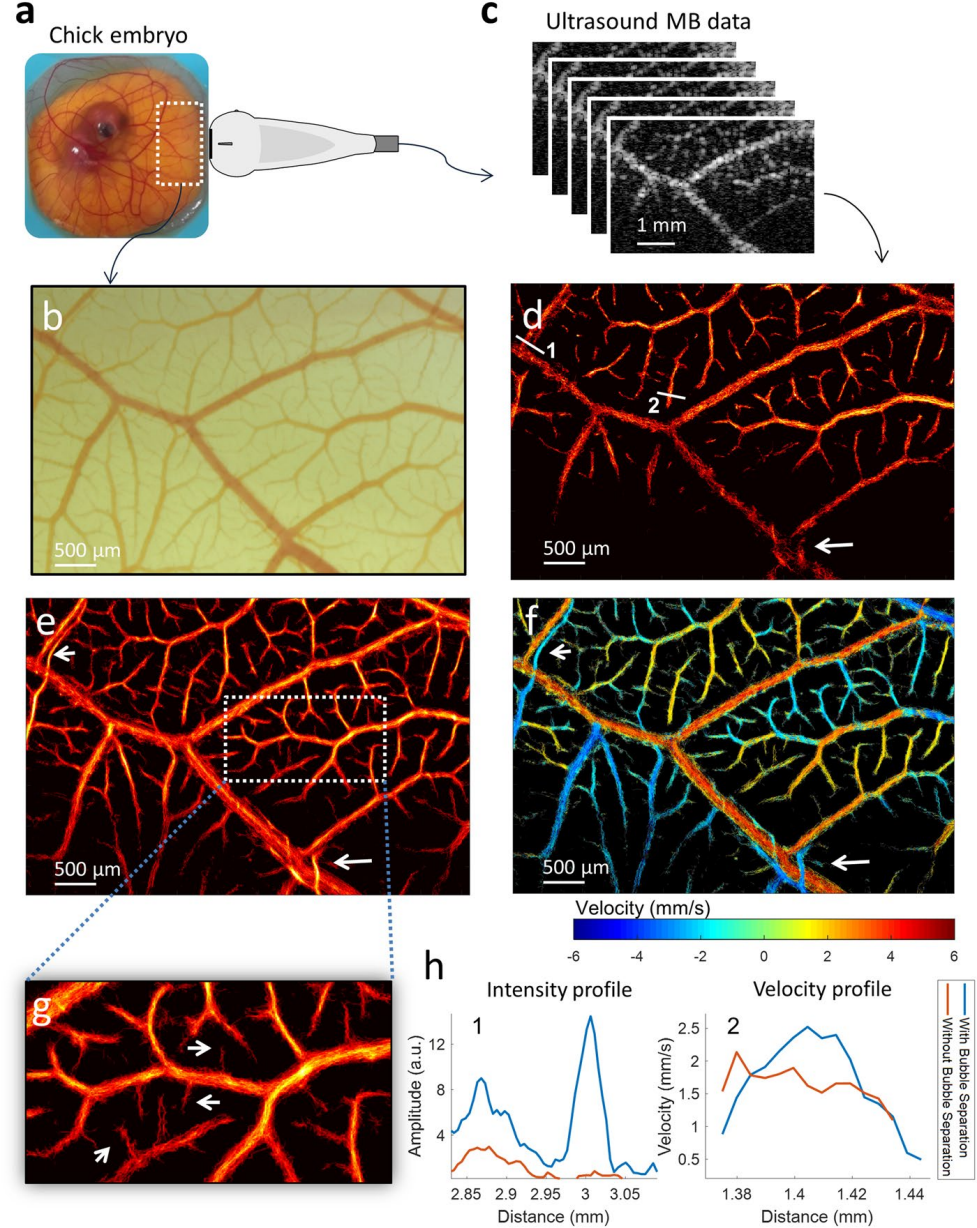
Chicken embryo CAM imaging with microscopic imaging validation. (**a**) An *ex ovo* chicken embryo imaged with a high-frequency transducer through an acoustic window in the side of container. (**b**) Co-registered optical imaging of the ultrasound field-of-view. (**c**) Ultrasound MB data was used to generate ULM super-resolution images. (**d**) Conventional ULM image reconstruction without microbubble separation. Lines 1 and 2 denote two manually selected vessel cross-sections of interest. (**e**) ULM intensity image with microbubble separation method. (**f**) ULM velocity map generated with microbubble separation. The arrows highlight adjacent vessels with opposing flow directions. (**g**) Magnified section of CAM with arrows pointing out microvasculature that was not detected by conventional ULM. (**h**) Intensity and velocity profiles of CAM vessels from manually selected line segments.

A chicken embryo CAM was used as a ‘living flow phantom’ for the purposes of validating the proposed method. ULM could be performed by opening an acoustic window into the side of the housing weigh boat and taking microscopic images from the top of the CAM (Fig. 4a). The *ex ovo* CAM provides a thin vascular bed with complex branching patterns and a minimal amount of tissue motion (Fig. 4b), making it an ideal model for optical imaging and ultrasound acquisitions. Super-resolution ULM, both with and without MB separation, was performed on a stack of 3600 ultrasound images (Fig. 4c) (corresponding to an acquisition time of 7.2 s) acquired from this model (Fig. 4d–f); both groups demonstrated vessels in the range of 240 µm to 10 µm and were in good agreement with the microvasculature depicted in the microscopic image (Fig. 4b). Compared to the MB-separated dataset (Fig. 4e), conventional ULM processing (Fig. 4d) provided fewer high-confidence MB tracks and therefore resulted in decreased saturation of the vascular bed during image accumulation. A substantially higher number of vessels in the near-capillary scale (10–30 µm in diameter) could be resolved following MB separation. These tiny vessels, indicated by white arrows in Fig. 4g, could not be detected by conventional ULM (Fig. 4d) within such a short acquisition time. Furthermore, ULM performance was degraded in situations where MB echo signals with different motion directions were overlapping and interfering, such as with intersecting vessels and densely packed capillary beds (indicated by the white arrow in Fig. 4d). Separation of these microbubbles into different subsets, and independent processing, allowed for accurate reconstruction of these intersecting and/or adjacent vessels, as shown in Fig. 4e,f (indicated by the arrows). Intensity profiles of two close-by vessels (as indicated by line 1 in Fig. 4d), obtained with and without microbubble separation, are depicted in Fig. 4h, and further highlight the ability of MB separation to avoid potential echo signal interference and yield improved recovery of microvasculature. Figure 4f depicts the velocity distribution of the ULM image obtained with the MB separation method. The velocity information is color-coded, with red indicating upward flow (positive) and blue indicating downward flow (negative) for better visualization; the distribution shows a reasonable velocity variation from ~6 mm/s at the larger vessel (240 µm) to ~1 mm/s at the smaller vessels (20 µm) in the CAM. Again, the capability to distinguish intersecting microvessels can be clearly demonstrated in the velocity map and a better parabolic distribution of the velocity across a microvessel (as indicated by line 2 in Fig. 4d) is also obtained by using the proposed method, which is expected for a vessel at this size (~70 µm, as shown in Fig. 4h). The velocity map for MB-separated ULM imaging (Fig. 4f) also demonstrated complex hemodynamic features, such as small blood vessels supplying the muscle layer of arterioles (as indicated by bottom white arrow in Fig. 4f), which could not be distinguished with conventional ULM imaging.

### Microbubble separation reduces acquisition time for microvessel imaging

**Figure 5:**
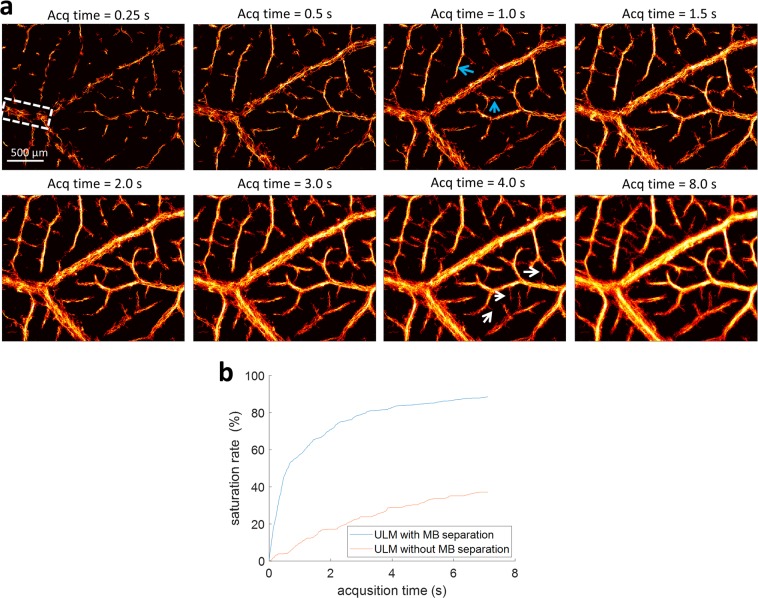
Influence of data acquisition time. (**a**) Super-resolved microbubble localization images of the CAM surface vasculature with incrementally increasing data acquisition lengths (accumulation time). Most of the microvessels, down to the ~10 µm diameter range, could be resolved within ~4 seconds using the MB separation technique. (**b**) The saturation rate of the vessel region indicated by the rectangle in Fig. 5a demonstrates that the vessel lumen was mostly populated within the first 2–3 seconds and plateaued beyond 4 s for the MB separation method, while for conventional ULM the saturation curve shows that the time to reach the plateau was much longer (>8 s).

A major challenge of super-resolution ultrasound microvessel imaging is that it requires a long acquisition or accumulation time to collect a sufficient number of isolated MB signals to fully populate microvasculature. With more MBs successfully detected and tracked at high MB concentrations, the acquisition time for full reconstruction of microvasculature can be considerably reduced with the proposed MB separation method. ULM images for the chick CAM obtained with different accumulation times are shown in Fig. 5a. Most microvessels at the ~50 µm range (indicated by blue arrows) have been reconstructed within 1 s of data length, while smaller branches at the 10 µm to 30 µm level (indicated by white arrows) are resolvable within 4 s of data length. Additional accumulated data length beyond 4 s for CAM imaging does not substantially increase the number of smaller vessels that were detected. The saturation rate of a vessel region (indicated by the rectangle in Fig. 5a) shows that the vessel lumen can be mostly populated within the first 2–3 seconds, and starts to plateau beyond 4 s, as shown in Fig. 5b. However, the method without MB separation requires a longer acquisition time to reach a saturation point (beyond 8 s) and may be unable to achieve the same number of detected microvessels due to heavily overlapping MB signals at high concentrations. These observations indicate that the acquisition time for data collection can be shortened to several seconds for reconstruction of microvasculature down to ~10 µm, which is within the breath-hold duration for human scanning and may be greatly beneficial for the clinical translation of ULM.

### Microbubble separation improved ULM performance for chick CAM tumor and brain

**Figure 6:**
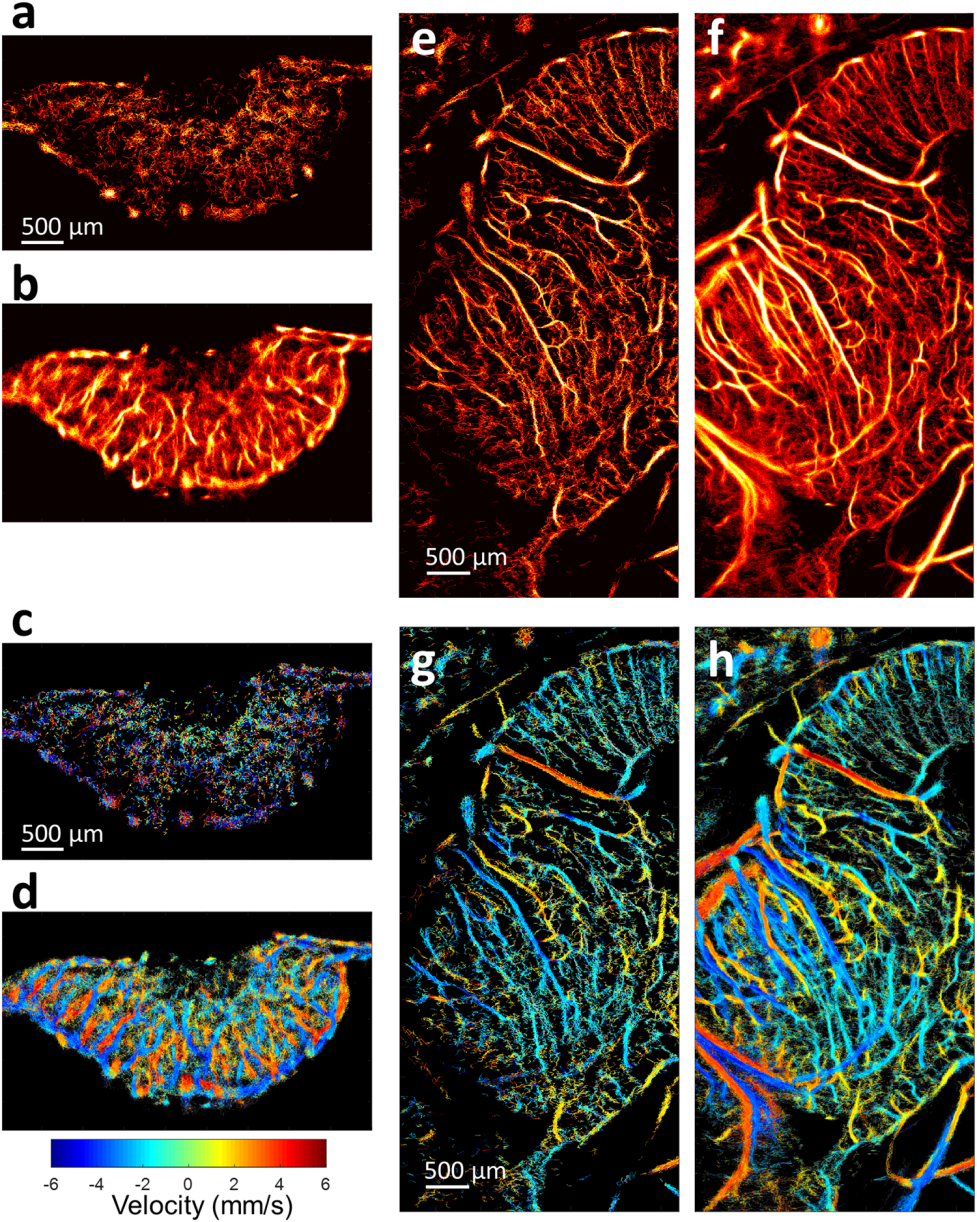
Application of proposed method to chicken embryo tumor and brain. ULM images were generated for CAM tumor both (**a**) without microbubble separation and (**b**) with microbubble separation. The corresponding images depict ULM velocity maps (**c**) without microbubble separation and (**d**) with microbubble separation. Likewise, ULM images of chicken embryo brains were generated (**e**) without microbubble separation and (**f**) with microbubble separation. As with the tumor data, the velocity maps (**g**) without microbubble separation were less populated than the velocity maps (**h**) with microbubble separation.

In order to test the proposed technique in more complex three-dimensional vascular networks, we applied MB separation to contrast-enhanced acquisition data acquired from tumor xenografts engrafted into the CAM and to datasets acquired from a chicken embryo brain. For the highly vascularized tumor model, localization and tracking of MBs are expected to be challenging, as MB signals tend to overlap within the same spatial tissue voxel—especially at the relatively high concentration of MBs used in this study. Furthermore, the complex hemodynamics of tumor tissue, with characteristically chaotic and intricate vascular topography, make accurate MB localization and tracking very difficult. Consequently, conventional ULM demonstrated a limited number of MB localizations within the tumor xenograft (Fig. 6a). In comparison, the application of MB separation was able to differentiate MB subpopulations within this complex vascular environment, thereby reducing the effective concentration of MBs within each filter range. This yielded a higher total number of localized MB centroids, and increased the amount of high-confidence MB trajectories within the acquisition, culminating in a high-quality super-resolution image (Fig. 6b). Likewise, velocity maps produced for the CAM tumor using conventional ULM were lacking definition, with few distinct blood vessels and/or velocities (Fig. 6c). With the application of MB separation, velocity maps showed well-defined flow directionality and clear vessel boundaries (Fig. 6d) and enabled differentiation of the neighboring feeding and draining vessels at high spatial resolution for a short period of acquisition time (data length of 7.2 s, corresponding to 3600 frames of ultrasound data used here).

Next, to test the performance in a hierarchically organized vessel bed, we imaged the brain of an EDD-18 (18^th^ day of embryonic development) chicken embryo through intact skull bone; a stack of 5000 frames of data (corresponding to data length of 16.6 s) was used to generate super-resolved images. As with tumor imaging, conventional ULM produced vascular localization maps (Fig. 6e) that were less populated than the corresponding MB-separated dataset (Fig. 6f). Highly detailed brain vasculature could be reconstructed with the proposed method (as shown in Fig. 6f, the full FOV image of the chick brain is shown in Sup Fig. 2) at high MB concentration and within a clinically relevant acquisition time. In particular, MB separation produced well-defined cortical microvasculature while also retaining a highly perfused and filled vessel lumen for cerebral arteries. This difference in performance was also evident in the velocity maps of the chicken brain. Conventional ULM produced thinner vasculature and had difficulty in regions where there were multiple flow velocity directions or where there were relatively larger vessels containing higher MB concentrations (Fig. 6g). MB separation allowed for better delineation in these complex flow velocity situations, producing velocity maps with rich hemodynamic information (Fig. 6h).

## Discussion

We have demonstrated that the proposed MB separation technique, which leverages the spatiotemporal characteristics of MBs in circulation to allow for independent analysis of sub-populations of MBs, improves the performance of MB localization and tracking for ULM imaging at high MB concentrations. This technique was tested *in vitro* using a flow channel phantom, as well as *in vivo* using an *ex ovo* chicken embryo model. We found that MB separation was superior at localizing MBs in a synthetically-combined flow channel dataset in comparison to conventional ULM, and could recover a well-defined parabolic velocity profile even under complex flow conditions (multiple flow rates and various flow directions). Likewise, MB separation applied to CAM imaging revealed better delineation of a broader spectrum of vascular bed features than conventional ULM alone (e.g.: multiple orders of blood vessels, ranging from capillary to arteriole), as validated with optical imaging. Furthermore, MB separation could produce velocity maps that retained complex flow patterning, such as the counter-directional blood flow of arterial capillaries (Fig. 4f**)**. Finally, the technique was applied to high-MB concentration contrast-enhanced ultrasound (CEUS) datasets acquired from multifaceted three-dimensional networks—namely the chicken CAM tumor xenograft model and chick brain. In both cases, MB separation produced well-defined microvascular features (with chaotic intratumoral capillary beds and cortical brain vasculature, respectively) while also retaining the highly perfused vessel lumen of feeding vasculature. Thus, the proposed technique offers a simple post-processing solution that alleviates the tradeoff between acquisition time and MB concentration for ULM imaging. This permits the widespread utilization of the method without the need to adjust experimental parameters and even allows for retrospective analysis of previously gathered datasets.

An underlying assumption of the proposed MB separation technique is that MBs will follow physiologically relevant blood flow trajectories and will therefore be constantly changing their relative positions and directions to one another. However, groups of MBs that are flowing in large vessels will exhibit high flow speeds and complicated flow dynamics, resulting in a low degree of correlation. In contrast, MBs in capillary beds will flow more slowly and stably and will maintain a high degree of correlation in their motion. These differences in movement speed, flow direction, and signal decorrelation produce tangible shifts in the spatiotemporal flow dynamics of MBs, which then correspond to distinct energy concentrations within different regions of the 3D Fourier domain (**k**-ω domain). With these assumptions in mind, we designed a set of conical **k**-ω domain filters to isolate MB subpopulations that are flowing at different speeds and trajectories. Applying this set of 3D cone-shaped filters to MB **k**-ω domain data separates the MB signal space into multiple subsets (15 subsets for *in vivo* data in this study) containing spatially sparser MB subpopulations with less flow complexity. We posit that this MB separation technique allows for useful ULM imaging at relatively high MB concentrations and is therefore preferable to the clinically relevant solutions of MB where MB signals may be spatially overlapping. Given that Hingot *et al*.^21^ report that the long acquisition times for ULM imaging are dictated by rare MB flow events in the microvasculature, it follows that higher MB injection concentrations would allow for reduced acquisition times. We observed that MB separation improved the performance of established MB localization and tracking algorithms^7^ and thereby resulted in better super-resolution images of vasculature.

Ultimately, the goal of the proposed MB separation algorithm is to produce multiple spatially sparse MB subsets from overlapping signals, thereby alleviating the need for dilution of indicator, rejection of overlapping MB signals, and long acquisition times. However, we acknowledge that our proposed method does not produce perfect MB separation and, indeed, such a goal may be impossible using only post-processing techniques. One salient concern with the proposed method is the risk of MB signal distortion resulting from splitting the MBs into two or more filters. To minimize the risk of MB signal distortion, we use relatively large passbands with smooth transitions and large overlaps between adjacent filters, which would avoid hard splitting of single MB signals. The remaining undesired signals can be further suppressed via intensity thresholding and correlation thresholding in the proceeding ULM processing steps (as detailed in Methods). As a result of filter overlap, MBs may be included within the passband of two or more filters and could therefore be localized and paired multiple times. This “MB doubling” effect would lead to an overestimate of vascular density value (i.e.: number of localized MBs) but should not influence the imaging the vasculature structure or morphology. Instead, “MB doubling” might be beneficial for maintaining the intensity of the ULM density image at the big vessel lumen, where the MBs signals would have been discarded without MB separation (as shown in Figs. 4d,e and 6e,f). Our observations comparing ULM imaging of CAM surface vasculature to optical images (Fig. 4) demonstrated that these concerns are perhaps unfounded, at least for relatively simple planar vascular beds. For three-dimensional vascular beds, we also showed that the judicious use of established MB pairing and tracking algorithms^7,22^ has produced well-defined vasculature for complex hemodynamic environments (Fig. 6). The number of separated datasets was 15 for the *in vivo* data in this study (13 subsets for the phantom data), but it should be noted that this number can be arbitrary, and the only requirement is to avoid significant MB signal smearing or distortion by using relatively large passbands. MB signals of each subset can be reviewed separately to empirically determine an appropriate passband for each filter. In general, a tradeoff exists between the number of separated datasets and computation cost. Although increasing the number of subsets can potentially provide a larger number of localized MBs, it also dramatically increases the computational cost. The optimal number of subsets and passband range would need to consider MB concentration(s), complexity of the flow dynamics for specific organ, flow speed range, and ultrasound center frequency, which would be varying from case to case.

This study has some limitations that should be addressed. The synthetic flow channel phantom data were combined under the assumption of purely linear interference, whereas an actual high concentration MB injection may have more complex and nonlinear interference characteristics. The purpose of using such a synthetic data was to artificially generate an extremely complicated MB flow dataset that contains different MB velocities and directions, in order to validate the capability of the proposed MB separation method in the differentiation of different MB sources. However, the synthetic combination of flow channel data produces conditions that are physiologically impossible, such as simultaneous counter-directional flow in a single vessel and MB boundaries that are truly spatially overlapping. We thus applied the proposed method to an individual flow channel data, which represented a realistic flow situation for a large vessel, and the results (Fig. 3) were in good agreement with that of the synthetic data. The proposed method was then tested *in vivo* using the *ex ovo* chicken embryo model, where the ground truth for microvascular structure and blood flow velocity are generally unknown. We relied on optical imaging to confirm our findings of microvascular structure in the planar CAM membrane; however, it should be noted that optical imaging, as implemented in this study, could not localize individual red blood cells and/or MBs to a high enough precision to confirm ULM measurements of capillary lumen and flow velocities. A gold-standard that could have been employed for the tumor and brain imaging results would be contrast-enhanced microCT using the Microfil contrast agent^23^; however, a pilot study found that injections of this indicator did not fully perfuse CAM vasculature. Microvessel flow velocity is difficult to validate *in vivo*, especially for deep three-dimensional vascular beds. Although the *ex ovo* chicken embryo offers an excellent exploratory model for ULM imaging, given that there is minimal tissue motion, limited attenuation, and shallow imaging depths, the use of this *in vivo* model potentially limits the direct translatability of the proposed technique for other models and clinical use. In particular, tissue motion is a substantial barrier to accurate super-resolution microvascular reconstruction. However, with numerous research groups proposing motion compensation techniques to correct for physiological motion in ULM imaging, tissue motion interference can still be mitigated^24–27^. The performance of the MB separation technique in more difficult imaging scenarios (severe respiratory motion, nonlinear acoustic propagation, MB nonlinearity and deep imaging targets) will be the subject of a future study.

In this paper we demonstrate a substantial improvement in the performance of ULM at relatively high MB concentrations by using a post-processing technique to separate MB subsets based on their spatiotemporal characteristics. The application of the proposed MB separation algorithm, which consists of a series of 3D cone-shaped filters in the **k**-ω domain, increases the possibility of observing spatially sparse MB events and therefore alleviates the need for MB dilution. This method could substantially reduce the acquisition times required for the reconstruction of microvasculature, which is critical for the clinical translation of ULM.

## Methods

### Microbubble separation algorithm

Assume $b({\bf{r}})$ to be MB signal as a function of the spatial coordinate **r**. A MB moving in a straight line with a constant velocity **v** (and speed $v=\Vert {\bf{v}}\Vert$) and initial position **r**_0_ at t = 0 can be described as:

1 $$g({\bf{r}},t)=b({\bf{r}}-{{\bf{r}}}_{0}-{\bf{v}}t)$$

The spatiotemporal Fourier transform of the moving MB can then be described as:

2 $$G({\bf{k}},\omega )=\iint g({\bf{r}},t){e}^{-j{\bf{k}}\cdot {\bf{r}}}{e}^{-j\omega t}d{\bf{r}}dt=\{\begin{array}{c}\begin{array}{cc}B({\bf{k}}){e}^{-j{\bf{k}}\cdot {{\bf{r}}}_{0}},when & {\bf{k}}\cdot {\bf{v}}+\omega =0\end{array}\\ \begin{array}{cc}0, & else\end{array}\end{array}$$

where *B*(**k**) is the Fourier transform of $b({\bf{r}})$, or can also be considered as the Fourier transform of the point spread function (PSF) of the ultrasound image. Equation (2) shows that the moving MB energy is concentrated on a 2D plane, as defined by:

3 $$\omega =-\,v{\bf{k}}\cdot \hat{{\bf{v}}}$$

inside the 3D Fourier domain (**k**-ω domain), where $\hat{{\bf{v}}}$ is the unit vector in the direction of ${\bf{v}}$. Consider that:

4 $$\Vert \omega \Vert =\Vert v{\bf{k}}\cdot \hat{{\bf{v}}}\Vert \le v\Vert {\bf{k}}\Vert $$

which indicates that the 2D MB planes from MBs moving at speed $v$ but in different directions form a space outside a cone-shaped surface defined by $\Vert \omega \Vert =v\Vert {\bf{k}}\Vert$ in the **k**-ω domain (An example of MB plane in the **k**-ω domain and the corresponding cone-shaped surface $\Vert \omega \Vert =v\Vert {\bf{k}}\Vert$ are indicated in Sup Fig. 3). Hence, by applying a high-pass cone filter with frequency response:

5 $$H({\bf{k}},\omega )=\{\begin{array}{cc}1 & \frac{\Vert \omega \Vert }{v}\le \Vert {\bf{k}}\Vert \\ 0 & else\end{array}$$

MBs with speed lower than $$v$$ can be preserved. Note that part of the MB signal with speed $$ > v$$ is included due to energy overlap in the **k**-ω domain, which can be partially suppressed in the subsequent ULM processing steps (such as thresholding and de-noising). Since MB signal is modulated by the center frequency (*f*_0_) of the transmitted ultrasound, its energy is centered at $${\bf{k}}=(0,\pm \frac{4\pi {f}_{0}}{c})$$, where *c* is the sound speed, deviating from the baseband in **k** domain. This facilitates the reduction of overlap between MBs with different speeds. Therefore, by applying a bandpass cone-shape filter with frequency response:

6 $$H({\bf{k}},\omega )=\{\begin{array}{cc}1 & \frac{\Vert \omega \Vert }{{v}_{2}} < \Vert {\bf{k}}\Vert \le \frac{\Vert \omega \Vert }{{v}_{1}}\\ 0 & else\end{array}$$

where ${v}_{2} > {v}_{1}$, MB energy spreading in the cone-shape range can be extracted. In the spatial frequency coordinates, this filter has the shape of a ring with inner and outer radii of $\frac{\Vert \omega \Vert }{{v}_{2}}$ and $\frac{\Vert \omega \Vert }{{v}_{1}}$, respectively. In practice, filters (such as the Butterworth filter) or window functions are applied to create smooth transition for $\frac{\Vert \omega \Vert }{{v}_{2}}$ and $\frac{\Vert \omega \Vert }{{v}_{1}}$ to avoid Gibbs ringing effects. It is also very important to allow a relatively large passband ($\frac{\Vert \omega \Vert }{{v}_{1}}-\frac{\Vert \omega \Vert }{{v}_{2}}$) to ensure dominant MB signals in this range do not distort after converting back to the spatial-temporal domain. To split the MB data into multiple subsets with different velocity ranges, as shown in Fig. 1, overlap of adjacent cone-shape filters is preferable, given the relatively large passbands. As a result, the same MB may appear in different subsets and be tracked for multiple times separately—a phenomenon we refer to as “MB doubling”. However, as we discuss below, this should not be a major concern for the final ULM image.

It should be noted that we are not separating all MBs moving with all possible velocities, which is very difficult due to the theoretical overlap of MBs in **k**-ω domain. Instead, we are extracting a group of MBs whose majority of spatiotemporal movement energies are concentrated inside the cone-shaped filter defined above, which may include energy from other MBs moving at other velocities. The hypothesis is that this undesired energy is suppressed inside the cone-shaped filter and, by subsequent post-processing like intensity thresholding and de-noising, this energy should be further reduced.

According to Eq. (3), the orientation of the 2D energy plane in the **k**-ω domain also indicates the direction of moving MBs, with those moving towards or away from the transducer allocated in distinct quadrants. Therefore, MBs extracted from each cone-shaped filter (Eq. 6) can be further divided into two subsets by applying a directional filtering. Equation (6) can thus be rewritten as:

7 $${H}^{+}({\bf{k}},\omega )=\{\begin{array}{cc}1 & \frac{\Vert \omega \Vert }{{v}_{2}} < \Vert {\bf{k}}\Vert \le \frac{\Vert \omega \Vert }{{v}_{1}},\omega {k}_{2} > 0\\ 0 & else\end{array}$$

8 $${H}^{-}({\bf{k}},\omega )=\{\begin{array}{cc}1 & \frac{\Vert \omega \Vert }{{v}_{2}} < \Vert {\bf{k}}\Vert \le \frac{\Vert \omega \Vert }{{v}_{1}},\omega {k}_{2}\le 0\\ 0 & else\end{array}$$

where ${H}^{+}({\bf{k}},\omega )$ and ${H}^{-}({\bf{k}},\omega )$ are filters for extracting MB signals moving towards and away from the transducer for the certain speed range, respectively; and ${\bf{k}}=({k}_{1},{k}_{2})$, where ${k}_{1}$ is the lateral direction perpendicular to the ultrasound beam, ${k}_{2}$ is the ultrasound beam direction.

An alternative and simplified MB separation method would be to take into account only the temporal information of the MB signals and apply a Fourier transform along the temporal dimension (slow-time dimension) of the MB ultrasound data. It is hypothesized that MBs with different velocities correspond to different frequency components in the temporal direction (i.e., Doppler frequency). The amount of Doppler frequency shift is proportional to MB moving velocity along the ultrasound beam direction, where a positive frequency indicates the MB moving towards the transducer, and a negative frequency indicates the MB moving away from the transducer. Therefore, the MB signal within a certain speed range can be extracted by applying a bandpass filter with frequency response as follows:

9 $${H}^{+}({\bf{r}},\omega )=\{\begin{array}{cc}1 & {\omega }_{1} < \Vert \omega \Vert \le {\omega }_{2},\omega  > 0\\ 0 & else\end{array}$$

10 $${H}^{-}({\bf{r}},\omega )=\{\begin{array}{cc}1 & {\omega }_{1} < \Vert \omega \Vert \le {\omega }_{2},\omega \le 0\\ 0 & else\end{array}$$

where ${H}^{+}({\bf{r}},\omega )$ and ${H}^{-}({\bf{r}},\omega )$ extract the positive and negative Doppler frequency components, respectively. Again, smooth transitions for ${\omega }_{1}$ and ${\omega }_{2}$ are typically applied via window functions or other filter designs to avoid the ringing effects of the Fourier transform. A relatively larger passband (${\omega }_{2}-{\omega }_{1}$) and larger overlap between adjacent filters would be preferable to avoid MB signal distortion when converting the MB data back to the spatial-temporal domain for ULM processing. This method only involves 1D Fourier transform and the corresponding filter design is relatively simple and straightforward, providing a fast implementation of MB signal separation alternative to the 3D Fourier transformation method.

### Flow channel imaging

A custom-made flow channel phantom (Gammex Inc., Middleton, WI, USA), with a 2-mm inner diameter straight flow channel which has an oblique angle of 30 degrees with respect to the surface of the phantom was used in this study. The outlet of the flow channel was connected to a syringe pump (Model NE-1010, New Era Pump Systems Inc., Farmingdale, NY, USA) that provided constant flow through the channel. Lumason MB suspension (Bracco Diagnostics Inc., Monroe Township, NJ, USA) was diluted with saline to approximately 1/2000 times the original concentration to provide a solution with adequately isolated MB signals. Ultrasound in-phase quadrature (IQ) data were acquired separately from the longitudinal view of the flow channel at four different flow rates (1.89 ml/min, 2.83 ml/min, 3.77 ml/min and 4.71 ml/min, corresponding to average flow speed of 10 mm/s, 15 mm/s, 20 mm/s and 25 mm/s, respectively, and corresponding to peak flow speed of 20 mm/s, 30 mm/s, 40 mm/s and 50 mm/s, respectively assuming parabolic flow profile) using a Verasonics Vantage ultrasound system (Verasonics Inc.,Kirkland, WA, USA) and a GE 9 L linear array probe (GE Healthcare, Wauwatosa, WI, USA). A 10-angle coherent compounding plane-wave imaging (step angle = 1°) was implemented for data acquisition with a center frequency of 6.25 MHz (single cycle pulse) for each transmit angle. The transmit voltage was lowered to 10 V (one-side voltage) to minimize MB destruction. The frame rate was 500 Hz, and for each flow rate setting, a stack of 6000 ultrasound frames (12 s) was acquired. For each flow rate, the flow direction was reversed by switching the outlet connected to the syringe pump, and the data acquisition was repeated. Thus, a total of 8 independent datasets were acquired (4 flow rates and two directions) at a relatively low MB concentration. All 8 IQ datasets were then linearly added frame-by-frame to generate a synthetic ultrasound dataset, which represents a complicated flow scenario at higher MB concentration, to validate the proposed MB separation method for ULM. The 8 independent datasets, each at low MB concentration, can also be processed separately to produce 8 ULM images, and the combination can be used as a reference standard.

### Ethics approval

No IACUC approval was required for the chicken embryo experiments presented in this paper, as under NIH PHS policy avian embryos are not considered to be live vertebrate animals.

### Cell culture

Renca cells (ATCC CRL-2947) were obtained from the American Type Culture Collection Inc. (Bethesda, MD) and cultured in accordance with ATCC guidelines. Growth medium was RPMI 1640 media (Wisent, QC) supplemented with 10% FBS (Hyclone, UT), non-essential amino acids (0.1 mM), sodium pyruvate (1 mM), and L-glutamine (2 mM). Cells were subcultured at a subcultivation ratio of 1:5 when >80% confluence. All cells were kept in a 37 ^o^C, 5% CO_2_, humidified incubator.

### Ex ovo CAM preparation

We followed the CAM preparation and tumor engraftment procedures outlined in^28,29^, with slight modification to facilitate co-registered acoustic-optical imaging. Fertilized chicken eggs were obtained from Hoover’s Hatchery (Rudd, IA) and placed into a turning humidified incubator (Digital Sportsman Cabinet Incubator 1502, GQF). For some preparations, acoustic windows were opened in the plastic weigh boat with a razor blade and sealed with saran wrap. On the fourth day of embryonic development (EDD-04) egg shells were opened with a rotatory dremel tool and contents were carefully transferred into the prepared weigh boats. Embryos were then transferred into a second humidified incubator (Caron model 7003-33) until ultrasound imaging on EDD-18.

### CAM tumor engraftment

Renca cells of high confluence (>80%) were trypsinized (0.05% Trypsin-EDTA, Wisent) and pelleted (centrifuged for 5 minutes at 300 g) on the day of engraftment (EDD-09). Matrigel (BD Bioscience) was combined with the cell pellet to obtain an inoculation dose of 4 × 10^5^ cells per 10 µL. This mixture was kept on ice until implantation into the CAM. On the engraftment day, EDD-09, a small disk of autoclaved Whatman No.1 filter paper was used to abrade the surface of the CAM, and 10 µL of the cell mixture was placed in the wound. Embryos were then returned to the incubator.

### Microbubble injection into the CAM

All CEUS imaging was performed using an intravenous injection of the commercially available Bracco Lumason microbubble solution. For this study, MBs were reconstituted with 1.0 mL sterile saline yielding a solution of approximately 1.8 × 10^9^ MBs/mL.

For CAM injection, an 18 G × 1.5-inch beveled needle tip was attached to 8 cm of Tygon R-3603 laboratory tubing. The open end of tubing was fitted with a glass capillary needle. With the aid of a dissecting microscope, the glass capillary needle tip was manually cannulated into a high-order vein on the CAM surface for contrast injection. A total volume of 70 μL of the MB solution was injected into each embryo.

### Ultrasound imaging of Chick Embryo

For CAM membrane and tumor imaging, a 15-angle compounding plane-wave imaging was performed (step angle = 1°) using a Verasonics Vantage ultrasound system equipped with an L35-16vX high-frequency linear array transducer (Verasonics Inc., Kirkland, WA) operating at a center frequency of 25 MHz. A two-cycle pulse was used for each transmit angle and the transmit voltage was set to 5 V (one-side voltage). The post-compounded frame rate was 500 Hz. For the CAM membrane, a total of 21 IQ datasets were collected from one bolus injection, with each dataset containing 720 ultrasound frames (corresponding to 1.44 seconds data length per dataset for the given frame rate). For each CAM tumor, a total of 5 acquisitions were gathered at 720 frames of IQ data each, for a total length of 3600 frames (7.2 seconds).

Chick embryo brain imaging was performed using a Vevo 3100 high-frequency ultrasound system (FUJIFILM VisualSonics Inc., Toronto, ON, Canada) equipped with an MX250S linear array transducer (FUJIFILM VisualSonics Inc.) transmitting at 21 MHz (single-cycle pulse). Line-by-line focused imaging was used to scan the chick brain through the intact skull bone with a 16 mm by 6 mm field-of-view (FOV) and one fixed transmit focus, leading to a frame rate of 301 frames per second (The full FOV image of the chick brain is shown in Sup Fig. 2). A total of 5 IQ datasets, each containing 1000 frames of ultrasound images, were acquired within one bolus injection of MBs in RF-mode at 1% transmit power, resulting in 5000 frames of data (corresponding to data length of 16.6 s) stored for post-processing.

### Optical imaging of CAMs

CAM optical imaging was performed using a Nikon SMZ18 stereomicroscope (Nikon, Tokyo, Japan) at 4x magnification, and images were digitized with an integrated Nikon DS-Ri2 digital camera (Nikon). Optical images were acquired from the top of the weigh boat housing the embryo, and an L35-16vX ultrasound transducer was placed to image the CAM surface through a lateral acoustic window in the side of the weigh boat (as illustrated in Fig. 4a). The field-of-view of the optical image was selected to correspond to the imaging plane of the ultrasound acquisition.

### Ultrasound data processing for ULM

A spatiotemporal SVD-based clutter filter was first applied to the ultrasound IQ dataset to extract moving MB signals by rejecting background tissue signals and stationary MB signals^7,30,31^. After tissue clutter rejection, a 3D Fourier transform was applied to convert the spatial-temporal MB signals into the **k**-ω domain. MBs with different speeds/directions correspond to energies concentrated in different regions of **k**-ω domain, as detailed above, which can then be split into multiple subsets of data with sparser microbubble concentrations using series of 3D cone-shaped filters. A 5^th^-order Butterworth filter was applied to the **k** space to create a smooth transition for $\frac{\Vert \omega \Vert }{{v}_{2}}$ and $\frac{\Vert \omega \Vert }{{v}_{1}}$ to minimize Gibbs effects for each 3D conical filter. Subsets of MB data with different speed ranges and directions were converted back to the spatiotemporal domain via an inverse 3D Fourier transform. Localization and tracking were then performed for each subset of MB data separately^7^ and the final ULM image was generated by combining separate ULM images from all the subsets, as shown in Fig. 1. For each subset of MB data, a similar ULM processing method was used as proposed in^7,22^. Specifically, noise equalization was first applied by dividing the filtered MB data by a noise intensity profile to equalize noise intensity throughout the imaging field-of-view^31^. The noise-equalized IQ data were then spatially interpolated to a desired resolution (4.9 μm for the Verasonics data, and 4.5 μm for the VisualSonics data) using a 2-D spline interpolation. Following interpolation, an intensity threshold was applied to the envelope of MB signals to remove the noisy background, which would also help suppress undesired energy leaking from other subsets of MBs. A point-spread function (PSF) specific to the imaging system and settings was derived from a multivariate Gaussian function. A 2-D normalized cross-correlation between each frame of the interpolated MB signal and the derived PSF was performed for MB localization. A threshold was applied to the cross-correlation map to remove pixels with correlation coefficient values lower than 0.6, which further suppresses unwanted signals from other MB subsets while preserving high confidence MB signals for the given subset. Then, MB centroids were localized using a regional maximum search of the cross-correlation peaks for each frame. A bipartite graph-based pairing and tracking algorithm was then applied to the identified MB centroids, with a minimum persistence of 7 frames^7,22^. A Kalman-filter-based algorithm was then applied to each MB trace to further reject unreliable MB tracks based on the MB movement acceleration and direction constraints, and improve in-painting of the MB trace and MB speed measurement^22^.

## Supplementary information

Supplementary information.

Supplementary Figure S1

Supplementary Figure S2

Supplementary Figure S3

## Data Availability

The data that support the findings of this study are available from the corresponding authors on request.
