# Supplementary material for: Short Acquisition Time Super-Resolution Ultrasound Microvessel Imaging via Microbubble Separation
Source: Sci Rep. 2020 Apr 7;10:6007. doi: 10.1038/s41598-020-62898-9 (PMC7138805; doi:10.1038/s41598-020-62898-9)
Supplement: Supplementary file 1 — Supplementary information. [file 41598_2020_62898_MOESM1_ESM.pdf]

Short Acquisition Time Super-Resolution Ultrasound Microvessel Imaging via Microbubble Separation

Chengwu Huang[1,§], Matthew R. Lowerison[1,2,3,§], Joshua D. Trzasko[1], Armando Manduca[4], Yoram Bresler[2,3,5], Shanshan Tang[1], Ping Gong[1], U-Wai Lok[1], Pengfei Song[1,2,3,*], Shigao Chen[1,*]

[1]Department of Radiology, Mayo Clinic College of Medicine and Science, Mayo Clinic, Rochester, MN,
[2]Beckman Institute, University of Illinois at Urbana-Champaign, Urbana, IL,
[3]Department of Electrical and Computer Engineering, University of Illinois at Urbana-Champaign, Urbana, IL,
[4]Department of Physiology and Biomedical Engineering, Mayo Clinic College of Medicine and Science, Rochester, MN,
[5]Coordinated Science Laboratory, University of Illinois at Urbana-Champaign, Urbana, IL
[§] These authors contributed equally to this work.

*Corresponding Authors:
Dr. Shigao Chen
Department of Radiology
Mayo Clinic
200 First Street SW
Rochester, MN 55905
Email: Chen.Shigao@mayo.edu

Dr. Pengfei Song
Department of Electrical and Computer Engineering
Beckman Institute for Advanced Science and Technology
University of Illinois at Urbana-Champaign
405 N. Mathews Ave.
Urbana, IL 61801
Email: songp@illinois.edu

Supplementary Figure Legends

Figure S1 | ULM images obtained from separate subsets of data from the flow channel phantom

ULM processing was performed on each subset of data extracted using the MB separation method, producing separate super-resolved images. (A) Super-resolved intensity images for the flow channel. (B) Super-resolved velocity magnitude image for the flow channel. The passband of the cone-shaped filter for each subset is indicated on the corresponding image. Notice that for each cone-shaped filter, the data was further split into two subsets with MBs moving towards or away from the transducer and processed separately.

Figure S2 | Full field of view images of the chick brain

(**A**) Ultrasound B-mode image of the chick brain. (**B**) Contrast-enhanced power Doppler image of the brain vasculature, which is subject to the diffraction limit of the ultrasound wave. (**C**) The proposed super-resolved ULM image with MB separation method.

Figure S3 | An example of MB plane and cone shaped surface in the k-ω domain

An example of the 2D plane in the **k**-ω domain of an MB moving in a straight line with a constant velocity **v** (and speed $v = \|\mathbf{v}\|$), obtained by applying 3D Fourier transform to a simulated RF data of a MB (The black plates represent the MB energy in the 2D plane). All the 2D MB planes from MBs moving at speed v but in different directions form a space outside a cone-shaped surface defined by $\|\omega\| = v\|\mathbf{k}\|$ in the **k**-ω domain (indicated on blue color).
