# Supplementary figures and images for: Short Acquisition Time Super-Resolution Ultrasound Microvessel Imaging via Microbubble Separation
Source: Sci Rep. 2020 Apr 7;10:6007. doi: 10.1038/s41598-020-62898-9 (PMC7138805; doi:10.1038/s41598-020-62898-9)

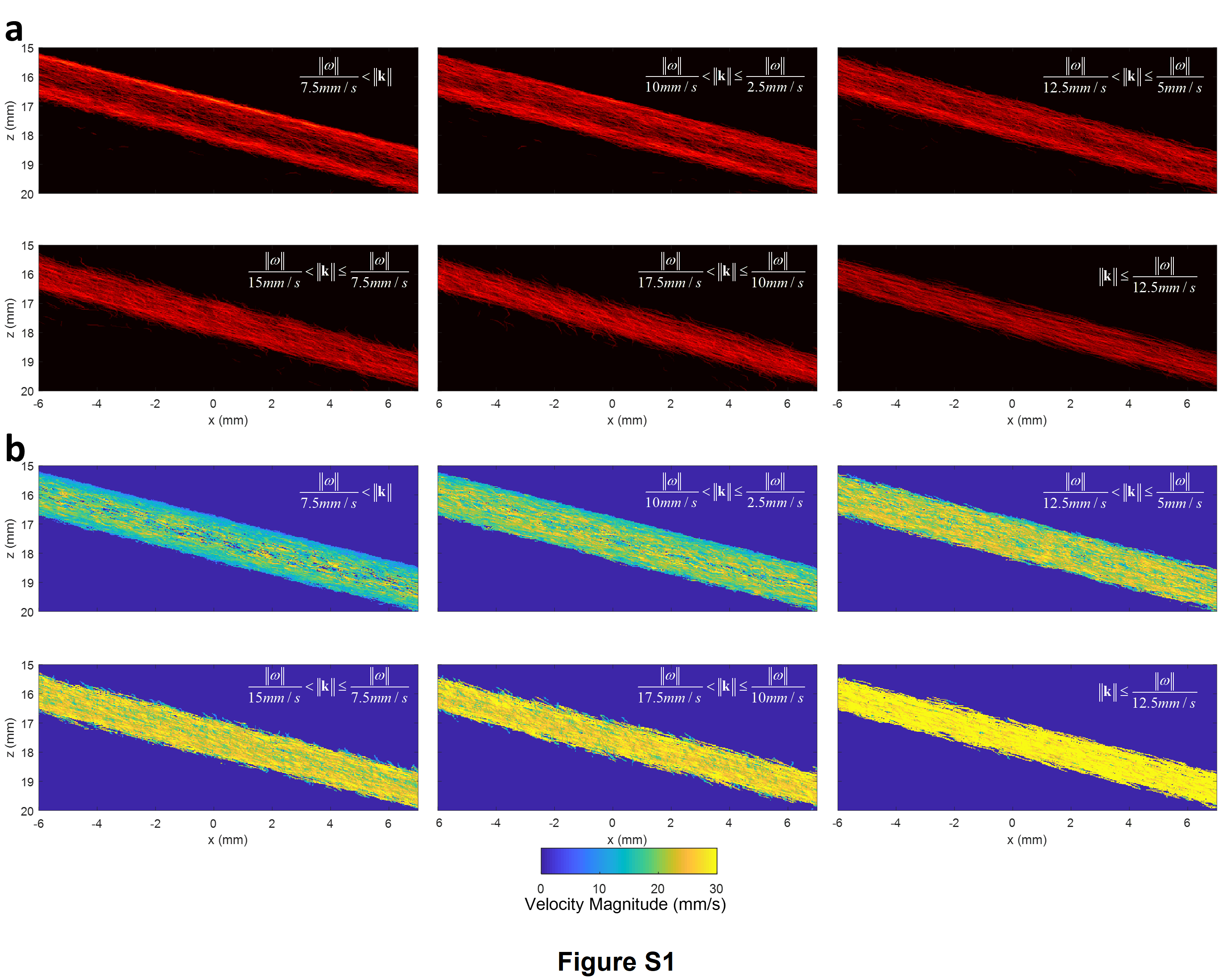

Supplement: Supplementary file 2 — Supplementary Figure S1 [file 41598_2020_62898_MOESM2_ESM.tif]

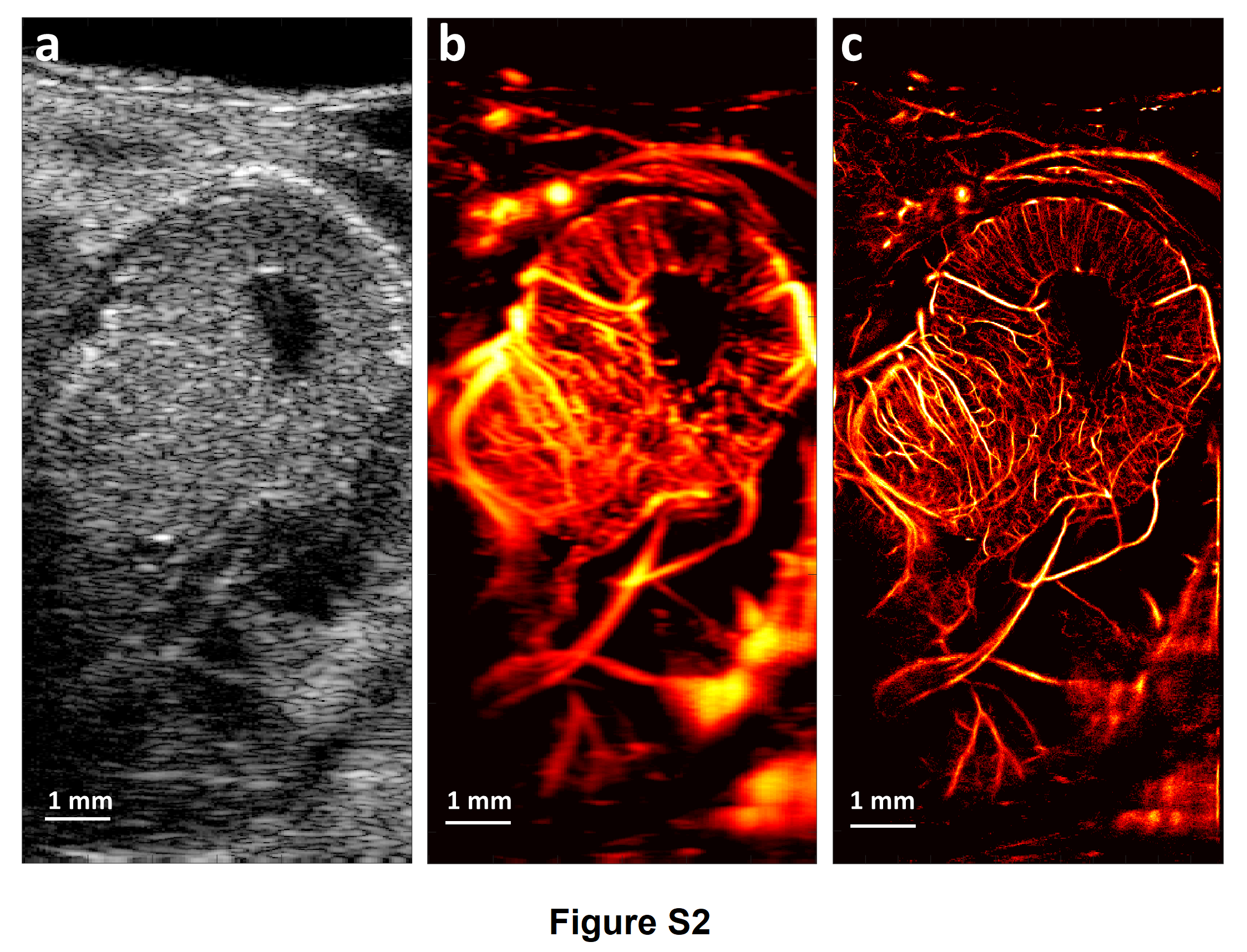

Supplement: Supplementary file 3 — Supplementary Figure S2 [file 41598_2020_62898_MOESM3_ESM.tif]

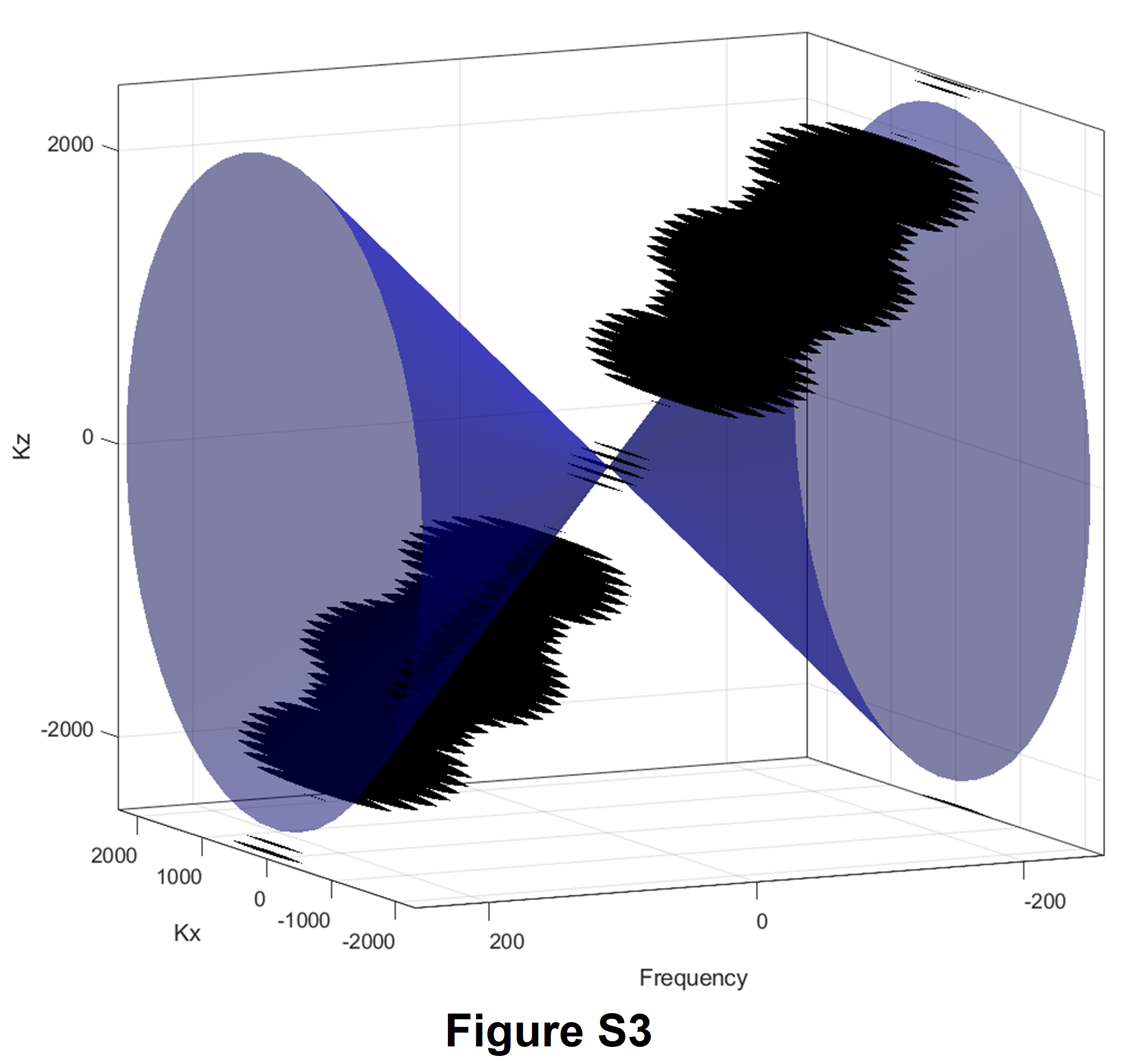

Supplement: Supplementary file 4 — Supplementary Figure S3 [file 41598_2020_62898_MOESM4_ESM.tif]
